# Electrically and Thermally Conducting Nanocomposites for Electronic Applications

**DOI:** 10.3390/ma3021478

**Published:** 2010-02-25

**Authors:** Wayne E. Jones, Jasper Chiguma, Edwin Johnson, Ashok Pachamuthu, Daryl Santos

**Affiliations:** 1 Chemistry Department, Binghamton University, Vestal Parkway East 13902, USA; E-Mail: johnso2@binghamton.edu (E.J.); 2 Materials Science and Engineering Program, Binghamton University, Vestal Parkway East 13902, USA; E-Mail: bj95108@binghamton.edu (J.C.); 3 Systems Science and Industrial Engineering Department, Binghamton University, Vestal Parkway East 13902, USA; E-Mail: apacham1@binghamton.edu (A.P.); santos@binghamton.edu (D.S.)

**Keywords:** nanocomposites, nanotubes, electrical conductivity, thermal conductivity, percolation threshold

## Abstract

Nanocomposites made up of polymer matrices and carbon nanotubes are a class of advanced materials with great application potential in electronics packaging. Nanocomposites with carbon nanotubes as fillers have been designed with the aim of exploiting the high thermal, electrical and mechanical properties characteristic of carbon nanotubes. Heat dissipation in electronic devices requires interface materials with high thermal conductivity. Here, current developments and challenges in the application of nanotubes as fillers in polymer matrices are explored. The blending together of nanotubes and polymers result in what are known as nanocomposites. Among the most pressing current issues related to nanocomposite fabrication are (i) dispersion of carbon nanotubes in the polymer host, (ii) carbon nanotube-polymer interaction and the nature of the interface, and (iii) alignment of carbon nanotubes in a polymer matrix. These issues are believed to be directly related to the electrical and thermal performance of nanocomposites. The recent progress in the fabrication of nanocomposites with carbon nanotubes as fillers and their potential application in electronics packaging as thermal interface materials is also reported.

## 1. Introduction

Heat dissipation in modern devices continues to be a challenge in the electronics industry as a result of continuous development of smaller, faster packages of integrated circuits. Finding the right combination of materials to fabricate thermal interface materials (TIMs) with high thermal conductivity has eluded researchers for a significant time and so continues to be a promising area of research. In this review, the history and properties of carbon nanotubes are reviewed in order to shed some light into their potential as polymer fillers, and the subsequent design and fabrication of nanocomposites for electrical and thermal transport applications is discussed.

## 2. Events Leading to the Discovery of Carbon Nanotubes

Nanocomposites are composed of fillers with nanoscale dimensions embedded in a polymer matrix. Different types of materials, such as silver particles, carbon fibers and boron nitride, for example, have been used over the years as filler materials in polymer matrices [1,2,3]. The discovery of multiwalled carbon nanotubes (MWCNTs) and the subsequent discovery of single-walled carbon nanotubes by Iijima in 1991 and 1993, respectively, have spurred the growth and development of carbon nanotube related research [4,5]. There has been significant growth in nanocomposites research since the first incorporation of carbon nanotubes in a polymer matrix by Ajayan and colleagues in 1994 in which carbon nanotubes were incorporated into an epoxy [6]. In line with the important synthesis-properties-application relationship it is essential to review the history and properties of carbon nanotubes in order to have an appreciation of the driving force behind the growth of nanocomposite related research.

**Figure 1:**
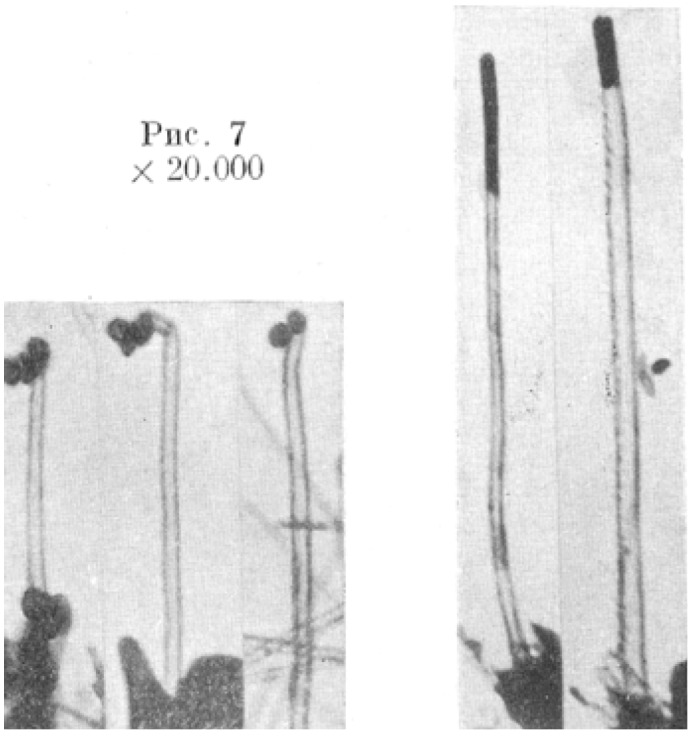
Hollow carbon fibers. Adapted from reference [7] with permission. Grobert, N. Carbon nanotubes – becoming clean. *Mater. Today* **2007**, *10* (1–2), 28–35.

In 1889 Hughes and Chambers announced the formation of carbon fibers followed by Schutzenberger and Schutzenberger making a similar report in 1890. The development of the Transmission Electron Microscope (TEM) by Siemens of which the first commercial version appeared in 1939 ensured that the structure of these fibers could now be elucidated. Radushkevich and Lukyanovich reported the formation of hollow carbon fibers in 1952 shown in Figure 1 [7].

**Figure 2:**
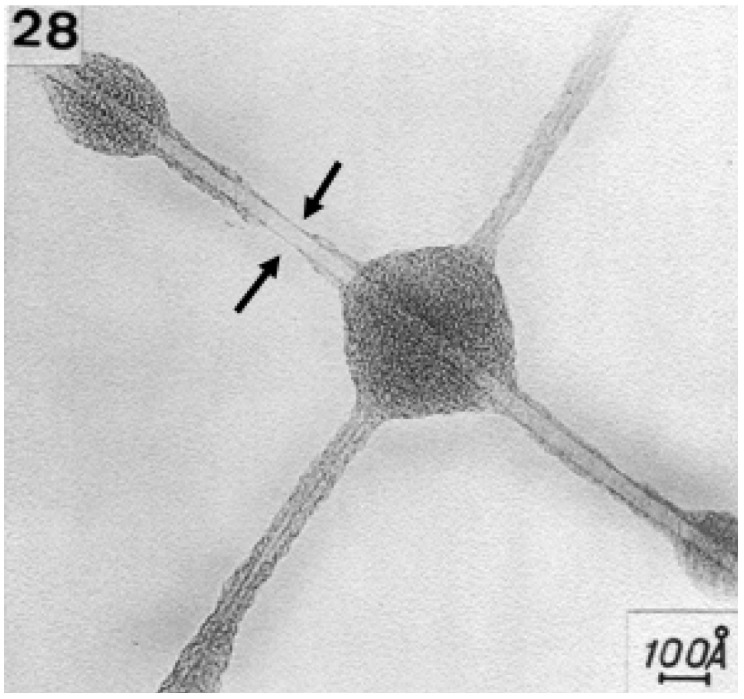
Carbon nanotube-like structures. Adapted from reference [8] with permission from Carbon. Kuznetsov, V.L. Who should be given credit for the discovery of carbon nanotubes? *Carbon* **2006**, *44*, 1621–1624.

This marked the initial success that would, decades later, lead to the discovery of carbon nanotubes. Figure 2 shows a viewgraph of structures that looked like carbon nanotubes reported by A. Oberlin, M. Endo and T. Koyama in 1976 [8].

## 3. Structure and Properties of Carbon Nanotubes

**Figure 3:**
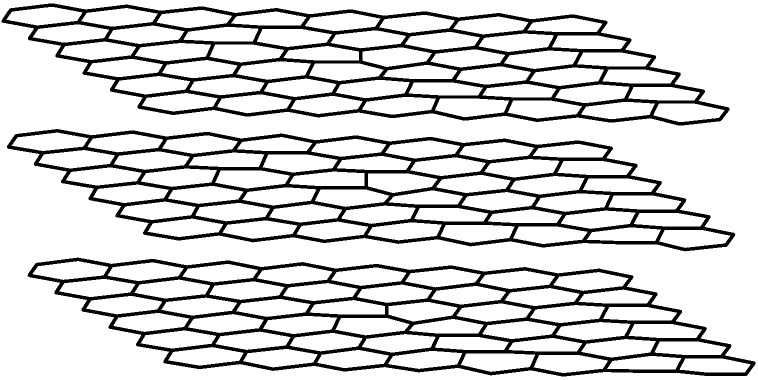
Structure of graphite layers.

The six electrons in the carbon atom are distributed in such a way that two of them occupy the 1s orbital and the remaining four electrons occupy the sp^3^ or sp^2^ as well as the sp hybrid orbital. These hybrid orbitals are responsible for the bonding in diamond, graphite, nanotubes or fullerenes. Each carbon atom in graphite has three outer-shell electrons occupying the planar sp^2^ hybrid orbital. The result is three in-plane σ bonds with an out-of-plane π bond and, consequently, a network of planar hexagons as shown in Figure 3.

The graphite networks, which are parallel to each other, have a spacing of 0.34 nm and are held together by weak van der Waals forces. The σ bond length is 0.14 nm with a bond energy of 420 kcal/mol in the sp^2^ orbital while in the sp^3^ configuration the bond length is 0.15 nm with bond energy of 360 kcal/mol. Graphite gets to be more thermally and electrically conductive because the out-of-plane π orbital is distributed over a graphite plane. Carbon nanotubes are formed by the rolling of graphite sheets with sp^2^ orbital as the predominant type of bonding [9].

Graphite can be converted to graphene by a variety of methods such as nanomechanical cleavage of graphite and chemical reduction of exfoliated graphite oxide. Graphene is a two-dimensional monolayer of sp^2^-bonded carbon atoms. It has been the focus of much research since its isolation because of its unique transport properties. There are efforts to use graphene as a transparent conductive electrode due to its high optical transparency. In comparison to traditional transparent electrodes, such as tin doped indium oxide (ITO), graphene films are characterized by high mechanical strength, flexibility and chemical stability. Graphene has also been used as filler material in polymer nanocomposites and there are arguments that since graphene can perform equally well as carbon nanotubes, it could be a better substitute to offset the possible toxicity and cost associated with carbon nanotubes.Transparent and flexible conducting materials are increasingly becoming popular in modern technologies, offering an important component of touch screens, video displays, and plastic solar cells. Besides the fact that indium is becoming increasingly expensive due to limited availability, ITO is relatively brittle, such that after repeated bending or strain, it cracks. Indium compounds are also relatively toxic. In order to address these technical problems, researchers are looking for alternative materials which are characterized by improved stable, environmentally friendly, high conductivity, good transparency and the ability to be processed in solution. The hybrid material of graphene and conducting polymers such as poly (3,4-ethylenedioxythiophene) (PEDOT), could provide a good alternative. The graphene-PEDOT hybrid material has been found to have good solution processabiltiy, excellent optical transparency, conductivity and flexibility [10,11,12,13].

**Figure 4:**
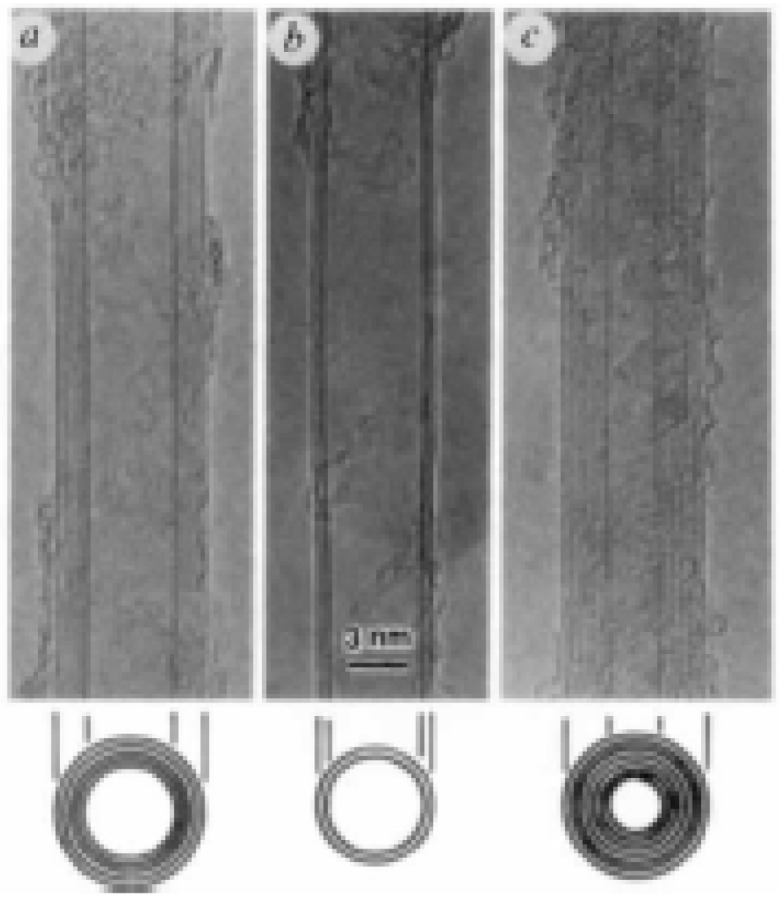
TEM of MWCNTs discovered by Iijima in 1991. Adapted from reference [4], with permission from Nature. Iijima, S. Helical microtubules of graphitic carbon. *Nature* **1991**, *354*, 56–58.

Although structures that looked like carbon nanotubes had been observed before, none of those discoveries stirred as much interest as the publication, ‘Helical microtubules of graphitic carbon’ in 1991 by S. Iijima in the journal *Nature*. The findings of Iijima resulted from the high resolution examination of soot produced during the synthesis of fullerene C_60_ using the Kraetschmer-Huffman method [14]. The carbon nanotubes were found to be MWCNTs. Their structures are shown in Figure 4.

**Figure 5:**
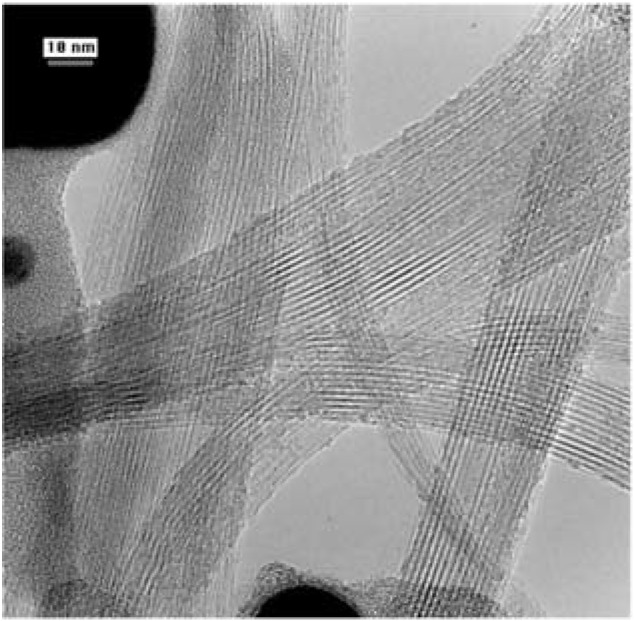
TEM of SWCNTs discovered by Iijima in 1993. Adapted from reference [15], with permission from Nature. Bethume, D.S.; Klang, C.H.; de Vries, M.S.; Gorman, G.; Savoy, R.; Vazquez, J.; Beyers, R. Cobalt-catalysed growth of carbon nanotubes with single-atomic-layer walls. *Nature*, **1993**, *363*, 605–607.

In 1993, Iijima and Ichihashi further reported the existence of single-walled carbon nanotubes (SWCNTs). Their structure is shown in Figure 5.

**Figure 6:**
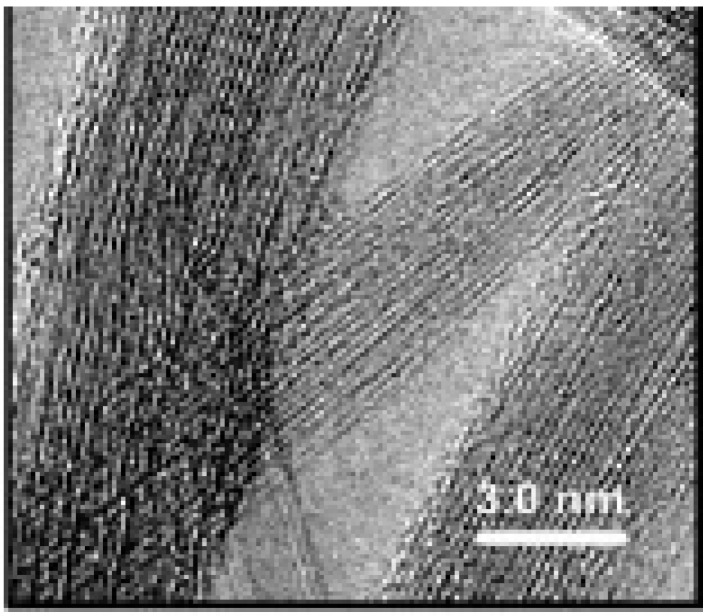
TEM of DWCNTs. Adapted from reference [16], with permission from Elsevier*.* Dai, H, Rinzler, A.; Nikolaev, P.; Thess, A.; Colbert, D.; Smalley R. Single-wall nanotubes by metal-catalyzed disproportionation of carbon monoxide. *Chem. Phys. Lett.* **1996**, *260*, 471.

In the same year, 1993, DS Bethune also produced SWCNTs using cobalt as the catalyst instead of iron as in the case of Iijima and Ichihashi [15]. In 1996, Dai and his colleagues reported the presence of a small number of double-walled carbon nanotubes (DWCNTs) along with single-walled tubes during the disproportionation of CO on alumina-supported Mo particles at 1200 °C. The presence or absence of DWCNTs was related to the nature of the catalyst used by Hafner and colleagues [16]. Figure 6 shows the structure of double-walled carbon nanotubes.

Today, carbon nanotubes are being produced by a variety of methods such as carbon arc discharge, laser–vaporization or laser ablation, chemical vapor deposition and electrolysis [17]. The production method tends to influence the properties of the resulting carbon nanotubes [18]. On average, some of the important properties of carbon nanotubes that have been reported are as follows: electron mobility of 10^5^ cm^2^/Vs, theoretical thermal conductivity of 6000 W/mK and a measured value of 3000 W/mK, modulus of 1 TPa and Tensile strength of 200 GPa [19,20]. Table 1 summarizes some of these properties [21].

**Table 1:** Properties of carbon nanotubes.

Carbon Nanotube Properties		
Property	SWCNTs	MWCNTs
Aspect Ratio	1000–10,000	1000–10,000
Electrical Conductivity	10^6^ S/m at 300K Individual	>10^5^ S/m
Electron Mobility	10^5^ cm^2^/Vs	10^5^ cm^2^/Vs
Thermal Conductivity	6600 W/mK Individual	3000 W/mK individual
Mechanical	Young’s Modulus: 1 TPa	Young’s Modulus: 1 TPa
	Tensile Strength: Up to 200 GPa	Tensile Strength: Up to 200 GPa

The high aspect ratio as a result of the nanometer sized diameter and micron sized length of these carbon nanotubes, coupled with the unique electrical, thermal and mechanical properties have resulted in scientists exploring how best to exploit them. Even though the application of individual carbon nanotubes is still to be exploited, bulk carbon nanotubes are attractive for application in, among others, electronics, sensing devices, and as fillers in polymer composites.

## 4. Nanocomposites

When polymers are reinforced with a network of fibers, polymer matrix composites are formed which are characterized by an improvement in tensile strength, stiffness, fracture toughness, abrasion resistance and corrosion resistance. Although polymers can be used as structural materials without reinforcement, their usage gets limited due to insufficient mechanical properties. Relatively low strength, coupled with low impact strength, is a characterization of polymer materials. Most of the commercial polymers are compounded with chemicals and/or other polymers to improve their usability or durability. Technically, compounding can be done to develop a polymer for a specific range of applications using carefully selected additives [22].

### 4.1. Insulating Polymers

Insulating polymers can be classified into two groups, namely, thermosets and thermoplastics. Thermoplastic resins become soft when heated, and may be molded or shaped while in a heated semifluid state. Thermosets are usually liquids or low melting point solids in their initial form. When used to produce finished goods, the thermoset resins are cured by use of a catalyst, heat or a combination of both. Once cured the thermosets cannot be converted back to their original liquid form. Unlike thermoplastics, cured thermosets will not melt and flow when heated and once formed they cannot be reshaped. The composites industry has been divided into thermoset and thermoplastic camps primarily because of differing requirements of their fabrication processes. Both types of plastic, however, benefit from reinforcement [23].

### 4.2. Conducting Polymers

**Figure 7:**
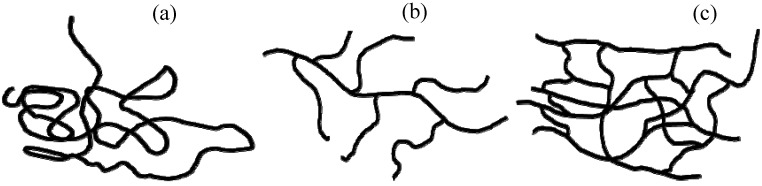
Macromolecular structure of polymers (a) Linear (b) Branched (c) Network.

A polymer is formed when a large number of small molecules, called monomers, link together in a repeated fashion. The word “polymer” is of Greek origin, with ‘poly’ meaning ‘many’ and ‘mer’ meaning ‘units’ [24]. By changing the chemistry involved, linear polymer chains can be linked together to form both branched and network structures as shown in Figure 7.

There are two main groups of polymers that have been used as polymer matrices in the synthesis of nanocomposites. These are non-conducting polymers and intrinsically conducting polymers. Examples of conducting polymers that have been commonly used in nanocomposite fabrication are polyaniline (PANi), polypyrrole (PPy) and poly(3,4-ethylene dioxythiophene) (PEDOT). Conducting polymer nanocomposites are commonly prepared by in-situ polymerization in which the monomer is chemically oxidized in the presence of the filler [25].

However, other methods such as direct mixing of the conducting polymer and carbon nanotubes as well as electrochemical polymerization have been used. The solution casting method is usually used for preparing nanocomposites of non-conducting polymers, although depending on the polymerization conditions, nanocomposites of certain polymers such as poly (methyl methacrylate) (PMMA) can also be prepared by *in situ* polymerization.

When fillers with diameters in the nanometer range are used to reinforce a polymer, the resulting blend of the nanofiller and the polymer is called a nanocomposite. The design and fabrication of nanocomposites for a particular application requires careful selection of the polymer matrix and the method of fabrication such as dissolving and casting, melt blend, in-situ polymerization and extrusion. For the dissolving and casting method it is important to select a suitable solvent that will dissolve the polymer and disperse the carbon nanotubes [26].

**Figure 8:**
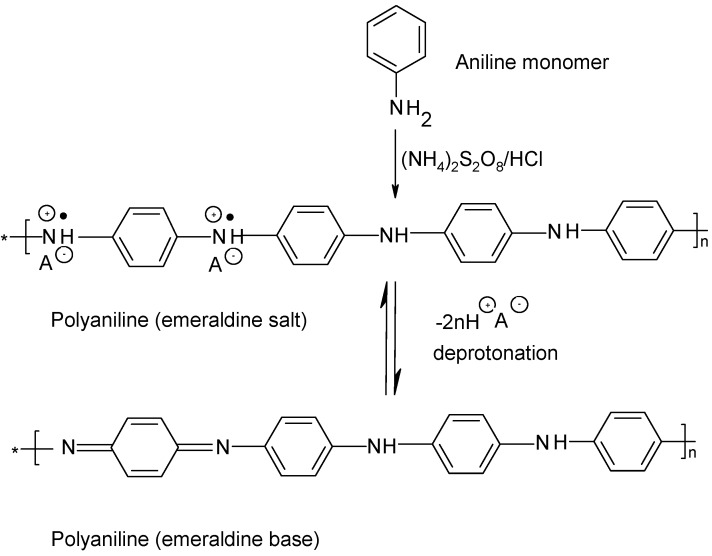
Formation of polyaniline from aniline and its two forms.

Figure 8 shows the structure of the two forms of PANi, the conducting doped form emeraldine salt and the non-conducting dedoped emeraldine base [27]. The formation of polyaniline results from the chemical or electrochemical oxidation of aniline.

**Figure 9:**
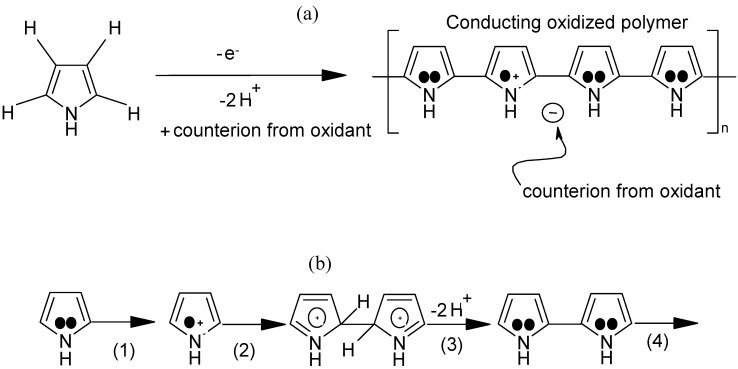
Mechanism illustrating the formation of polypyrrole (a) Overall process (b) Proposed mechanism: 1. monomer oxidation; 2. dimerization; 3. proton loss; 4.repetition.

Pyrrole, a five member ring, can be polymerized chemically and electrochemically as well, leading to the formation of polypyrrole. The scheme in Figure 9 illustrates the mechanism by which the chemical oxidation of pyrrole occurs by the loss of an electron to form radicals which dimerize followed by proton loss and the subsequent formation of polypyrrole [28].

**Figure 10:**
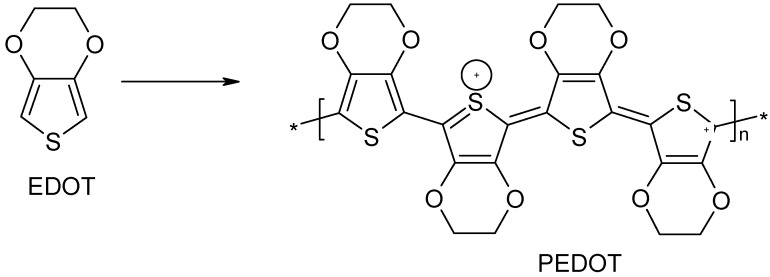
Formation of PEDOT.

The formation of PEDOT [29] from the monomer EDOT follows the same mechanism as in pyrrole whereby the formed EDOT radicals dimerize, followed by proton loss and the subsequent formation of PEDOT as shown in Figure 10. PEDOT is an intrinsically conducting polymer that can be used as an active material in flexible organic electronics due to its remarkably high conductivity, transparency and environmental stability. It suffers, however, from being insoluble, which limits its application in devices. One alternative has been the use of PEDOT combined with the water-soluble poly (styrenesulfonic acid) (PSS). However, PEDOT/PSS is characterized by low conductivity. This is why graphene, which is highly conductive, could be a better additive for PEDOT which would offer a graphene-based hybrid material with high conductivity, transparency and flexibility [30].

**Figure 11:**
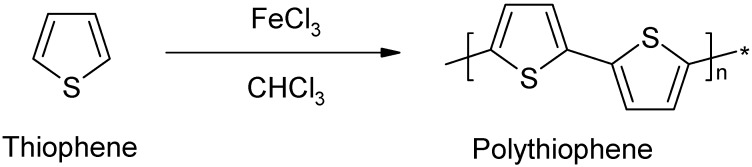
Formation of Polythiophene.

Another important intrinsically conducting polymer that has been used as a matrix for nanocomposite fabrication is polythiophene. Polythiophenes are conjugated polymers which form some of the most environmentally and thermally stable materials. Polythiophene materials can be used as electrical conductors, nonlinear optical devices, polymer LEDs, electrochromic or smart windows, photoresists, antistatic coatings, sensors, batteries, electromagnetic shielding materials, artificial noses and muscles, solar cells, electrodes, microwave absorbing materials, new types of memory devices, nanoswitches, optical modulators and valves, imaging materials, polymer electronic interconnects, nanoelectronic and optical devices and transistors. Polythiophene, like many polyaromatic compounds is insoluble in organic due to the presence of the rigid backbone. In order to make it soluble, substitution in the 3 and/or 4 position is necessary. Polythiophene can be synthesized at room temperature by in situ polymerization using ferric chloride (FeCl_3_) as an oxidant in chloroform (CHCl_3_). Polythiophene loaded with fillers such as carbon nanotubes can therefore be prepared by in situ polymerization just like polypyrrole, polyaniline and poly (3,4-ethylenedioxythiophene) nanocomposites. Figure 11 shows the formation of polythiophene from the thiophene monomer [31,32,33].

While there is growth and interest in the field of nanocomposites, challenges abound. Among them are the dispersion of carbon nanotubes in the polymer matrix, the nature of the polymer/filler interface, its thickness and how it affects load transfer from the polymer matrix to the carbon nanotubes, junction resistance, slippage in MWCNTs and in bundles of SWCNTs [34]. Load transfer is how a structure distributes the forces of a load over the supporting structural members. It depends on the interfacial shear stress between the filler and the matrix. Shear stress is the stress applied parallel or tangential to a face of a material. A high interfacial shear stress will transfer the applied load to the fiber over a short distance, while low interfacial shear stress will require a long distance. The three main mechanisms of load transfer from a matrix to a filler are micromechanical inter-locking, chemical bonding and weak van der Waals forces. Micromechanical interlocking is usually made difficult by the atomically smooth surface of carbon nanotubes. Unless the carbon nanotubes are functionalized the probability of chemical bond between the matrix and the carbon nanotubes is low [35].

The advantage of the excellent mechanical and electrical properties of carbon nanotubes can be exploited if good interfacial interaction is achieved [36]. When fillers are embedded into polymer matrices there is going to be a zone where the surfaces of the fillers and matrices get to interact. The nature of this zone between the filler surface and the matrix depends on the microstructure characteristics of the fillers as reinforcement, in which any of the three mechanisms, physical interaction, physical-chemical interaction and mechanical interlocking, may be dominant and the van der Waals forces of attraction are the primary binding forces at the interface [37]. Although there are many other materials that have been used as fillers, the advantage of carbon nanotubes that make them likely to improve the interface is their nanometer sized diameter. The result is interaction at the molecular scale, thereby minimizing the areas with no interfacial contact. Ideally, it is expected that when properly dispersed in a polymer matrix, carbon nanotubes should form a percolating network through which electrons can flow from one end of the nanocomposite to the other [38]. Where carbon nanotubes are dispersed randomly conduction is expected to be isotropic. As the filler content is increased in a polymer matrix, there is a gradual transition from the predominant polymer properties to the properties of the filler as indicated by the parameter being measured be it electrical, thermal or mechanical. However, this is not always the case since even when initially dispersed, the carbon nanotubes tend to settle upon casting. The transition point at which the filler properties start to be dominant is referred to as the percolation threshold, thus a percolating network or continuous path is formed from one end of the material to the other when the number of carbon nanotubes per unit volume of polymer exceeds the threshold value [39]. With better dispersion, a random network is likely to be formed easily, making the threshold lower.

**Figure 12:**
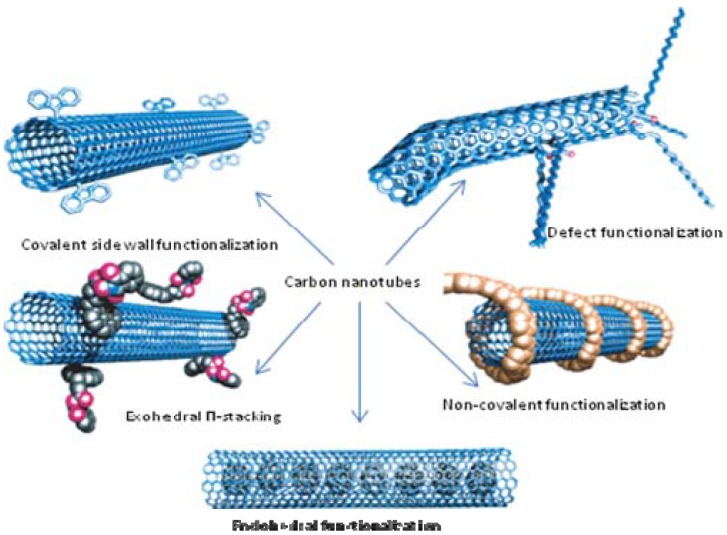
Functionalization methods. Adapted from reference 36, with permission from the publisher. Wagner, H.D.; Vaia, R.A. Nanocomposites: issues at the interface. *Mater. Today* **2004**, *7* (11), 38–42.

In order to overcome the problem of dispersion, different methods have been employed. Among them are bath sonication, tip sonication, oxidation and functionalization and use of surfactants [40]. Although useful, these methods have their problems such as the shortening of carbon nanotubes and unwanted defect creation. While oxidation generally introduces oxygen atoms as carbonyl and carboxylic groups, functionalization may result in unwanted functional groups that may not convert into part of the desired product, but becomes an impurity. To date, the different ways of functionalization that have been reported are non-covalent exohedral functionalization with polymers, defect group functionalization, non-covalent exohedral functionalization with molecules through π-stacking, sidewall functionalization and endohedral functionalization with fullerene C_60_ [41]. Figure 12 illustrates the results of these functionalization methods.

Good dispersion has been observed when carbon nanotubes are dispersed in the presence of cationic surfactants such as cetyltrimethylammonium bromide (CTAB) and other nonionic surfactants [42]. Some surfactants such as CTAB can be eliminated from the final product during washing which means it does not become an impurity. However, there are surfactants which do not wash away and therefore become an impurity in the nanocomposite.

In non-conducting polymers, even when conducting fillers are well dispersed, there are always going to be portions of the nanocomposite that are insulating. In fact, the more dispersed the carbon nanotubes, the greater the amount of insulating polymer separating the carbon nanotube strands, which complicates the idealized percolation threshold at the smallest possible loading of carbon nanotubes. In intrinsically conducting polymers, electrical conductivity has been reported to occur through three mechanisms namely intra-chain, inter-chain and inter-particle [43]. The presence of the already conducting polymer and the conducting fillers will therefore ensure the presence of conductivity domains throughout the nanocomposite. When carbon nanotubes are dispersed in a non-conducting polymer matrix, they become wrapped with a thin layer of insulating polymer. This thin layer is likely to make inter-chain conduction difficult, yet in a conducting polymer the thin layer that wraps the nanotubes is conducting.

**Figure 13:**
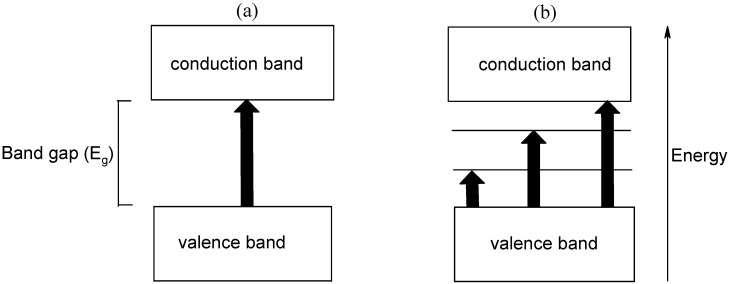
Effect of doping on the band gap and energy in conducting polymers. (a) an undoped and (b) an oxidatively doped (bipolaron) conducting polymer.

Conducting polymers fit in the category of semiconducting materials. Their electrical conductivity can best be explained in terms of band gap, whereby for semiconductors with a small band gap the energy gap between the conduction band or highest occupied molecular orbital (HOMO) and the valence band or lowest unoccupied molecular orbital (LUMO) is small. When foreign atoms (dopants) are introduced in a polymer the number of charge carriers is increased, coupled with the formation of a charge transfer complex. The result is the formation of highly delocalized radicals. Cationic radicals are formed from acceptor dopants while anionic radicals result from dopants that donate electrons. Oxidative doping results in the formation of new low energy transitions due to bipolaron production and, consequently, two new states within the gap as illustrated in Figure 13. The polarons and bipolarons are mobile along the polymer chain and therefore charge transport is by mobile polarons and bipolarons as well as by hopping electrons. It is believed that charge transfer occurs between carbon nanotubes and the conducting polymer matrix [44,45].

The inherent properties of carbon nanotubes after production are essential to the final properties of the nanocomposite in which they are incorporated. Differences in the properties of carbon nanotubes produced by arc-discharge and catalytic methods have been reported. While catalytic methods are capable of being tailored to produce aligned patterns essential for uniform electrical properties in nanocomposites, the degree of graphitization is low which compromises their mechanical and electronic properties and consequently those of the nanocomposites. Carbon nanotubes produced by arc-discharge are characterized by a high degree of graphitization, and excellent mechanical and electrical properties [46].

Despite the challenges that have been discussed, nanocomposites are still thought to hold the key to solving a myriad of problems in electronics, medicine, defense and aerospace as evidenced by work and patents filed related to electrochemical capacitors, sensors, conductivity and photoconductivity, photovoltaic cells and photodiodes, optical limiting devices, solar cells, Schottky diodes, high-resolution printable conductors, electromagnetic absorbers and transistors. In the electronics industry, heat dissipation has been a problem for a significant time and researchers still think there can be improvement on the materials that are used to dissipate heat from electronic devices such as computers, cell phones, and others [47].

## 5. Electrical and Thermal Conductivity of Nanocomposites

A number of reports have been made on the electrical and thermal conductivity of nanocomposites at different filler loadings using different fillers and polymer matrices. Table 2 shows the electrical percolation threshold of some of the nanocomposites that have been synthesized over the years. Table 3 summarizes the thermal conductivity measurements for selected nanocomposites.

**Table 2:** Percolation threshold of carbon nanotube nanocomposites. Adapted with permission from reference [18], Du, J.H.; Bai, J.; Cheng, H.M. The present status and key problems of carbon nanotube based polymer composites. *eXPRESS Polym. Lett*. **2007**, *1* (5), 253–273.

Author	Sample	Percolation threshold [wt %]
Sandler *et al.*	Epoxy + CNTs	0.0025 - 0.004
Sandler *et al.*	Epoxy + aligned MWCNTs	0.0025
Allaoui *et al.*	Epoxy +CNTs	0.5 – 1
Shaffer *at al.*	PVA + MWCNTs	5 – 10
Dufresne *et al.*	PS + MWCNTs	≤3
Andrews *et al.*	PS + MWCNTs	0.25 vol %
Barraza *et al.*	PS +SWCNTs	8.5
Zhang, *et al.*	HDPE + SWCNTs	4
McNally *et al.*	PE + MWCNTs	7.5
Hu *et al.*	PET + MWCNTs	0.9
Seo *et al.*	PP + CNTs	1 - 2
Seo *et al.*	PP + CNTs	2
Tchmutin *et al.*	PP + SWCNTs	4.5 vol %
Meincke *et al.*	PA6 + CNTs	4 – 6
Coleman *et al.*	PmPV + CNTs	8.5
Kymakis *et al.*	P3OT + SWCNTs	11

**Table 3:** Thermal conductivity of selected nanocomposites.

Author	Sample	Thermal Conductivity [W/mK]
H. Huang *et al.* [48]	Silicone elastomer + 0.4 wt % MWNTs	Aligned: 0.65, Dispersed: 0.31
H.Fakushima *et al.* [49]	Nylon 6 + 20 vol % xGnP (graphite)	>4
C. H. Liu *et al.* [50]	Silicone elastomer + 3.8 wt % SWCNTs+MWCNTs	65% enhancement
P. C. Song *at al.* [51]	Silicone elastomer + 1.6wt % MWCNTs	1.59
C. H. Liu *et al.* [52]	polydimethylsiloxanerubber + 2wt % MWCNTs	0.190
J. E. Peters *et al.* [53]	PS + 30wt % MWCNTs	>0.6
M. B. Bryning *et al.* [54]	Epoxy+1wt % SWCNTs	80% enhancement
R. Haggenmueller *et al.* [55]	HDPE + 30wt % SWCNTs	3.5
M. J. Biercuk *et al.* [56]	Epoxy + SWCNTs	125% enhancement
F. Du *et al.* [57]	Epoxy + SWCNTs	0.61

Table 2 reveals that the minimum amount of carbon nanotubes required to form a percolating network that facilitates electrical conductivity in the nanocomposites is considerably low. Apart from the type of polymer matrix and the type of carbon nanotubes, the most dominant factor that seems to influence the percolation threshold is the alignment of the carbon nanotubes. The lowest percolation threshold in Table 2 of 0.0025 wt % was recorded for an aligned MWCNT-loaded epoxy nanocomposite. The thermal conductivity data in Table 3 shows that carbon nanotube loaded nanocomposites have increased thermal conductivity compared to the corresponding polymers which usually have a thermal conductivity of 0.2 W/mK. [58] The data also show a significant increase in thermal conductivity when graphite nanofibers are used as fillers. At a loading of 20 vol % a thermal conductivity value of greater than 4 W/mK was achieved. Even though there is evidence of improvement in thermal and electrical conductivity, the values are still considerably below those predicted by theory.

A number of models have been put forward that try to predict thermal conductivity in nanocomposites. The most basic models are the rule of mixtures and the inverse rule of mixtures. The rule of mixtures model (or series model) is the weighted average of matrix and filler thermal conductivities. This model works well with unidirectional composites with continuous fibers. The inverse rule of mixtures (parallel model) usually underestimates the thermal conductivity of short fiber composites. The more sophisticated heat transfer models can be categorized either as heat flux law models, where the temperature field is solved for an assumed geometry, or Ohm’s law models based on electrical series resistance analogy. An example of an Ohm’s law model is the Nielsen model. It is a very versatile model for conductive short fiber/particulate systems. It accounts for almost all important factors including the constituent thermal conductivities, volume fraction, aspect ratio, orientation, and packing of the filler material. The model is based on the analogy of equations describing the elastic moduli of a composite and the version for thermal conductivity uses the versatile Halpin-Tsai equations as its basis. [58] The Maxwell–Eucken and the Agari models have also been used to determine the thermal conductivity of nanocomposites. [59] Another possible reason that might contribute to this disparity is the type of nanotubes and their purity as well as the presence of defects [60,61,62].

**Table 4:** Electrical Conductivity of Polycarbonate (PC)/MWCNTs nanocomposites.

Sample	Percolation Threshold (wt %)	Electrical Conductivity S/cm
PC/dispersed MWCNTs	~5	2.08 × 10^-3^
PC/ dispersed MWCNTs	~2	2.87 × 10^-5^
PC/dispersed MWCNTs	~5	3.65 × 10^-4^
PC/dispersed MWCNTs	~5	5.69 × 10^-6^
PC/dispersed MWCNTs	~0.1	8.14 × 10^-5^

Our recent work on the fabrication and characterization of nanocomposites for electrical and thermal conductivity has shown the dependency of these properties on the type of polymer matrix, the post-production treatment, and the dispersion method [63]. Table 4 and Table 5 show some examples of the polymer nanocomposites that were fabricated in our group. The data include their percolation threshold and thermal conductivity. In Table 4, the first four samples were made from polycarbonate and the same type of MWCNTs that had received different post-production treatments. These MWCNTs were produced by a combustion process. The percolation threshold for one sample is around 2 wt % while that of the other three start at around 5 wt %. Interestingly, the electrical conductivities vary as well, showing the effect of post-production treatment on percolation threshold and electrical conductivity of nanocomposites. The last sample in Table 4 was made from polycarbonate and a different type of MWCNT that was manufactured by chemical vapor deposition. The percolation threshold was found to be around 0.1 wt % and the electrical conductivity was 8.14 × 10^-5^ S/cm.

**Table 5:** Thermal Conductivity of Epoxy/MWCNTs nanocomposites.

Sample	Thermal Conductivity (W/mK)
Epoxy	0.20
Epoxy/0.1 wt % MWCNTs (dispersed)	1.85
Epoxy/ 0.1 wt % MWCNTs (dispersed)	1.91
Epoxy/0.1 wt % MWCNTs (dispersed)	1.55

The effect of different post-production treatments on the thermal conductivity of nanocomposites can be seen in Table 5. The MWCNTs that were used in this case were the ones that were produced by the combustion process except that they received different treatment after production. At the same percentage loading of 0.1 wt %, the nanocomposites were found to have different thermal conductivity values.

## 6. Conclusions

This brief survey of the current status of conducting nanocomposites serves to illustrate the progress that has been made in this field since the inception of this type of research in the nineties. Although there are still challenges associated with the fabrication of nanocomposites such as dispersion and alignment of carbon nanotubes, existing data illustrate that their electrical and thermal conductivity values make them ideal candidates for use as TIMs as well as in those areas were electrically conducting pads are needed. Research efforts are focused in finding better ways to transfer the high thermal and electrical conductivity of carbon nanotubes to the polymer matrix in the nanocomposites. The current thermal and electrical conductivity values that have been reported are still very low compared to those of pristine carbon nanotubes. Nevertheless, there is hope that polymer/carbon nanotube nanocomposites might hold the key to solving the heat dissipation problem associated with the miniaturization of electronic devices.
